# When Pacing Fails After Generator Replacement: A Stepwise Diagnostic Approach to a Reversible Lead–Header Interface Problem

**DOI:** 10.3390/reports9020137

**Published:** 2026-04-29

**Authors:** Fulvio Cacciapuoti, Antonietta Buonomo, Salvatore Crispo, Massimo Russo, Ciro Mauro

**Affiliations:** 1 Division of Cardiology “Antonio Cardarelli” Hospital, 80131 Naples, Italy; 2 Department of Cardiology “Pellegrini” Hospital, 80134 Naples, Italy

**Keywords:** pacemaker malfunction, generator replacement, loss of capture, lead malfunction, unipolar pacing, device interrogation

## Abstract

**Background and Clinical Significance**: Early loss of pacing capture after pacemaker generator replacement is an uncommon but potentially life-threatening event, especially in pacemaker-dependent patients. In this setting, device malfunction is often initially attributed to intrinsic lead damage, prompting consideration of invasive lead revision or extraction. However, not all early failures reflect true structural lead dysfunction. Careful interpretation of device interrogation findings, particularly in relation to pacing configuration, may uncover reversible causes and support a more targeted diagnostic and management approach; **Case Presentation**: A 61-year-old man with complete atrioventricular block presented with recurrent syncope six days after elective pacemaker generator replacement. The electrocardiogram showed absence of effective ventricular pacing with a slow escape rhythm. Device interrogation revealed loss of ventricular capture in bipolar configuration associated with markedly elevated impedance, initially raising concern for lead malfunction. However, switching to unipolar pacing restored effective capture with normal electrical parameters, suggesting preserved lead integrity and prompting reconsideration of the underlying mechanism. Further diagnostic evaluation, including imaging and intraoperative assessment, was therefore undertaken to clarify the cause and guide management; **Conclusions**: Early pacing failure should not automatically be equated with lead damage. Beyond documenting a reversible lead–header interface problem, this case highlights the diagnostic value of a stepwise approach integrating pacing configuration behavior, targeted imaging, and intraoperative header-independent testing. Such an approach may facilitate rapid localization of reversible defects and help avoid unnecessary lead revision.

## 1. Introduction and Clinical Significance

Pacemaker generator replacement is a common and generally safe procedure, with a relatively low incidence of major complications in contemporary practice [1]. Nevertheless, early device malfunction, although uncommon, may have serious clinical consequences, particularly in pacemaker-dependent patients, in whom loss of effective pacing can rapidly lead to syncope or hemodynamic compromise.

When pacing failure occurs shortly after generator replacement, the most immediate concern is intrinsic lead dysfunction, including conductor fracture, insulation failure, or exit block [2]. This interpretation is clinically relevant, as it may prompt consideration of invasive lead revision or extraction, procedures that are associated with non-negligible risks and are guided by established consensus recommendations [3,4].

However, not all early pacing abnormalities reflect true structural lead damage. Device interrogation plays a central role in this setting, and the combined assessment of pacing thresholds, sensing, and impedance, particularly when interpreted in relation to pacing configuration, can provide important insights into the underlying mechanism of malfunction [5]. Configuration-dependent discrepancies have been previously described, including unexpected loss of bipolar pacing with preserved unipolar function, suggesting disruption of the electrical circuit rather than distal lead failure [6,7].

Abnormalities at the lead–header interface, such as incomplete lead insertion or impaired connector contact, represent a recognized cause of pacemaker malfunction [8]. Although relatively uncommon, these mechanical issues may mimic more serious lead-related complications and can lead to misdiagnosis if not specifically considered [9]. Additional reports have highlighted how defects within the connection system may present with atypical or misleading device behavior, further complicating the diagnostic process [10,11].

In this context, a structured diagnostic approach is essential. Rather than relying on isolated device parameters, integrating clinical presentation, configuration-dependent interrogation findings, and targeted imaging may allow accurate localization of the problem and identification of reversible causes.

Clinical significance: We report a case of early loss of ventricular capture after generator replacement in which a stepwise diagnostic workflow, guided by pacing configuration behavior, led to the recognition of a reversible lead–header interface abnormality and avoided unnecessary lead revision [3,12].

## 2. Case Presentation

A 61-year-old man with a history of complete atrioventricular block treated with a dual-chamber permanent pacemaker implanted in 2016 presented to the emergency department with recurrent dizziness and syncope. Six days earlier, he had undergone elective pacemaker generator replacement with a Medtronic Azure S DR MRI SureScan generator (model W3DR01), while the original leads were preserved (Medtronic CapSure V MRI ventricular lead and Medtronic CapSureFix atrial lead). The generator replacement had been performed approximately 9 years after the initial pacemaker implantation.

His medical history included chronic ischemic heart disease treated with triple coronary artery bypass grafting, chronic lymphocytic leukemia, and type 2 diabetes mellitus. His chronic medications included antiplatelet therapy, statins, and beta-blockers. He was not receiving Bruton tyrosine kinase inhibitors or other therapies known to affect atrioventricular conduction [13]. At presentation, he was hemodynamically stable but markedly bradycardic.

**Figure 1:**
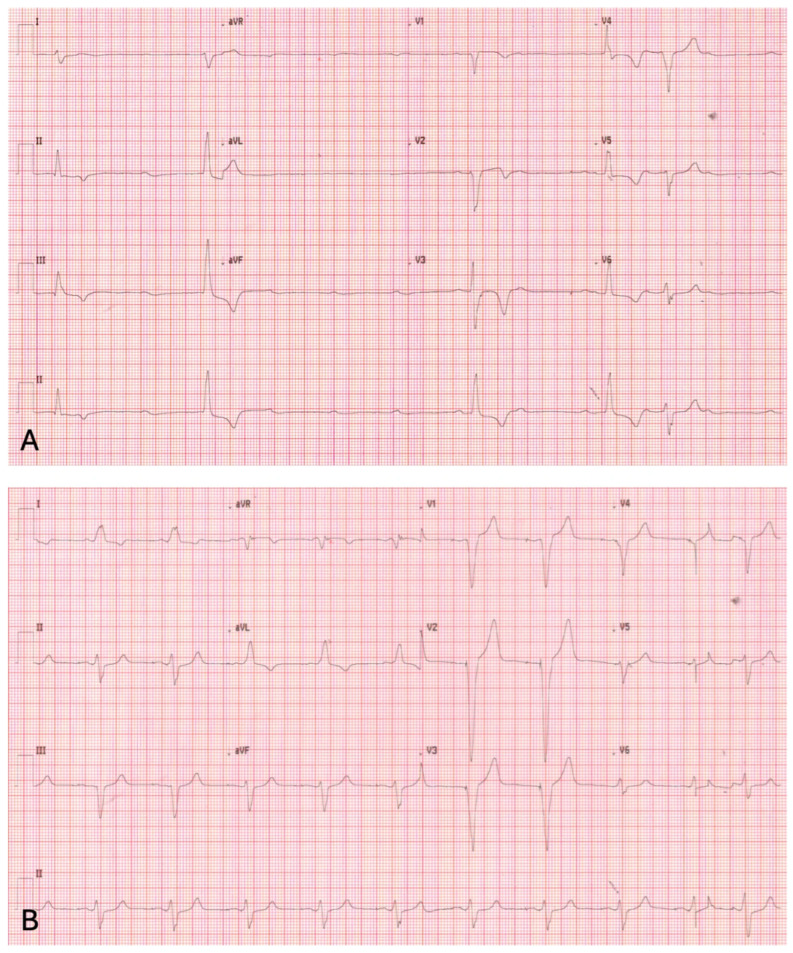
(**A**) Surface 12-lead electrocardiogram obtained at admission showing complete atrioventricular block with a slow ventricular escape rhythm and absence of effective ventricular pacing. (**B**) Surface 12-lead electrocardiogram after surgical revision demonstrating restoration of effective ventricular pacing with regular paced QRS complexes.

Surface electrocardiogram showed complete atrioventricular block with a ventricular escape rhythm at approximately 30 beats per minute and absence of effective ventricular pacing (Figure 1A). Given his pacemaker dependency and marked bradycardia, low-dose isoproterenol infusion was used as a short-term bridge while urgent definitive management was arranged, under continuous monitoring given the potential risk of ventricular arrhythmias in this setting. No ventricular arrhythmias occurred during pharmacological support.

Device interrogation revealed loss of ventricular capture despite DDDR programming. Ventricular lead impedance was markedly elevated in bipolar configuration (>3000 Ω), raising initial concern for lead malfunction. However, switching to unipolar pacing immediately restored reliable ventricular capture, with normalization of impedance values, suggesting preserved distal lead integrity.

In the days preceding admission, the patient had experienced additional transient syncopal episodes and had already returned to the implanting center for evaluation, where device interrogation had reportedly not demonstrated a clear abnormality. During our assessment, gentle manipulation of the generator within the pocket did not reproducibly affect capture or impedance values. There was no history of recent chest trauma, vigorous movements of the ipsilateral arm, or magnetic resonance imaging between generator replacement and presentation.

**Figure 2:**
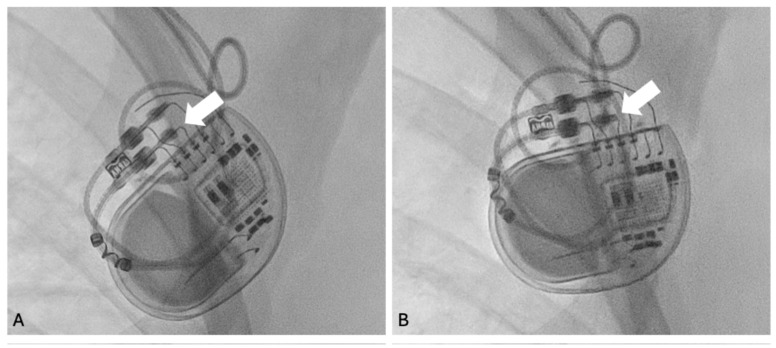
Fluoroscopic images of the pacemaker generator showing the ventricular lead–header interface. (**A**) Incomplete insertion of the ventricular lead connector pin into the pacemaker header (arrow), resulting in selective loss of bipolar pacing. (**B**) Correct lead seating after surgical pocket revision, with the connector pin fully advanced into the header (arrow) and restoration of normal bipolar pacing function.

The configuration-dependent findings raised suspicion of a proximal lead–header interface abnormality. Fluoroscopic evaluation in anteroposterior (AP) and left anterior oblique (LAO) projections demonstrated incomplete insertion of the ventricular lead connector pin into the device header (Figure 2A).

The patient subsequently underwent surgical pocket revision under local anesthesia. After reopening the pocket and exposing the generator, visual inspection confirmed incomplete seating of the ventricular lead connector pin. The set screw was loosened using the manufacturer’s torque wrench, and the lead terminal pin was inspected, without evidence of structural damage.

The ventricular lead was then tested directly using an external pacing system analyzer (PSA), bypassing the device header. This confirmed normal lead function, with a pacing threshold of 0.6 V at 0.4 ms, sensed R-wave amplitude of 12.5 mV, and bipolar impedance of 510 Ω.

The connector pin was then fully advanced into the header until complete seating was achieved, and the set screw was securely retightened. Repeat intraoperative testing confirmed stable bipolar function.

Following revision, effective ventricular pacing was restored (Figure 1B), with normalization of electrical parameters, fluoroscopic confirmation of proper lead connector pin seating within the header (Figure 2B), and complete resolution of symptoms.

At 1-month follow-up, the patient remained asymptomatic, with stable bipolar sensing, pacing threshold, and impedance values and no recurrence of syncope.

## 3. Discussion

Early pacemaker malfunction after generator replacement is clinically significant, particularly in pacemaker-dependent patients, because it may immediately suggest intrinsic lead failure and thereby lower the threshold for invasive reintervention [2,3]. In this setting, the main clinical challenge is not simply to confirm device malfunction, but to identify its mechanism accurately enough to distinguish true lead damage from a reversible connection problem [5,11]. Although several key reports describing configuration-dependent pacing abnormalities and lead–header interface issues date back to earlier decades, these observations remain clinically relevant given that the basic design and electrical principles of contemporary pacing systems have largely remained unchanged [5,12].

In the present case, the decisive clue was the configuration-dependent discrepancy between bipolar and unipolar pacing. This finding is consistent with fundamental concepts of cardiac pacing physiology, as bipolar pacing depends on electrical continuity between the tip and ring electrodes, whereas unipolar pacing relies on a circuit between the tip electrode and the device can. Accordingly, a defect involving the proximal ring pathway at the lead–header interface may abolish bipolar capture and produce markedly elevated bipolar impedance while preserving effective unipolar pacing [6,7,11]. While similar configuration-dependent abnormalities have been reported previously, the present case illustrates the practical value of this pattern, showing how it can be integrated with procedural timing, targeted fluoroscopy, and header-independent lead testing in a stepwise diagnostic pathway that localizes the defect and avoids unnecessary lead intervention [6,7,8,12].

The intermittent nature of the malfunction is also clinically relevant. Incomplete insertion of the connector pin may create unstable electrical contact, particularly at the ring-header interface. Small changes in generator position, arm movement, tissue tension, or respiratory motion may then transiently improve or worsen electrical continuity. This may explain why symptoms and interrogation findings can be intermittent in lead-header connection abnormalities [11,12].

A stepwise diagnostic workflow is particularly useful in this setting [5,12]. The close temporal relationship with generator replacement initially suggested a procedure-related mechanism. Device interrogation confirmed malfunction, but not its precise location. Comparison of pacing configurations further narrowed the differential diagnosis by suggesting preserved distal lead integrity. The main diagnostic issue was to distinguish true lead failure from other causes of early pacing failure (Table 1) [2,5,12].

**Figure 3:**
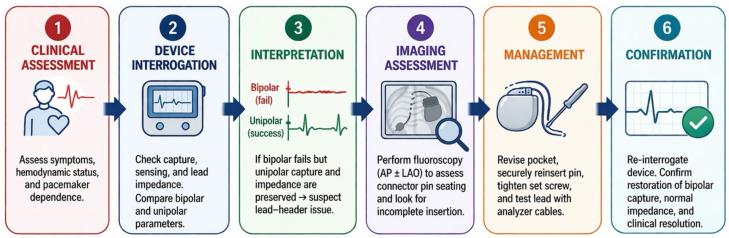
Stepwise diagnostic–management approach to early loss of ventricular capture after pacemaker generator replacement. AP = anteroposterior; LAO = left anterior oblique.

Although abrupt loss of bipolar capture with markedly elevated impedance may raise concern for conductor fracture, insulation defect, or other intrinsic lead dysfunction, the configuration-dependent pattern observed here made distal structural lead failure less likely. Immediate restoration of ventricular capture with unipolar pacing suggested preserved distal tip function and myocardial excitability, arguing against generalized exit block. This interpretation was further supported by direct intraoperative testing with an external PSA, which bypassed the device header and showed normal pacing threshold, sensing, and bipolar impedance. Lead dislodgement was also less likely, since normal bipolar function was restored after correction of the connector abnormality without lead repositioning [2,12]. Taken together, the pacing configuration findings, fluoroscopic evidence of incomplete connector insertion, and header-independent lead testing localized the defect to the lead–header interface and avoided unnecessary lead revision or extraction (Figure 3) [3,11].

An important practical implication is that impedance should not be interpreted in isolation. Abrupt impedance abnormalities may indeed raise concern for lead failure, but device diagnostics are most informative when interpreted together with capture behavior, sensing, configuration dependence, and procedural timing [5,12]. In particular, restoration of capture after switching to unipolar pacing should prompt immediate reconsideration of distal structural lead failure and redirect attention toward the connector system (Table 2) [6,7,11].

## 4. Conclusions

Early loss of pacing capture after generator replacement should not be automatically assumed to reflect intrinsic lead failure. In this patient, preserved unipolar capture provided an important clue to a reversible lead–header connection problem.

Careful interpretation of device interrogation, supported by fluoroscopy and intraoperative lead testing, allowed the cause to be identified and corrected promptly, avoiding unnecessary lead revision.

## Figures and Tables

**Table 1:** Main causes of early pacing failure after pacemaker generator replacement and typical diagnostic clues.

Cause	Typical Diagnostic Clues
Lead–header interface abnormality	Early post-replacement loss of capture, configuration-dependent abnormalities, abnormal impedance, incomplete connector seating
Intrinsic lead damage	Abrupt impedance abnormality, loss of capture, possible sensing abnormalities
Exit block/threshold rise	Loss of capture despite preserved lead continuity, often with normal impedance
Lead dislodgement	Loss of capture, altered sensing, lead position change
Pulse generator malfunction	Device dysfunction not explained by lead or connector findings
Programming-related cause	Resolved by reprogramming, without structural abnormality

**Table 2:** Comparison of ventricular lead electrical parameters obtained intraoperatively, at clinical presentation, and after pocket revision, showing selective bipolar pacing failure with preserved unipolar function due to a lead–header interface abnormality.

Parameter	Configuration	At Presentation	Intraoperative	After Pocket Revision
Pacing threshold (V @ 0.4 ms)	Bipolar	Loss of capture	0.6	0.7
	Unipolar	0.8	Not assessed	0.8
Sensed R-wave amplitude (mV)	Bipolar	Not measurable	12.5	12.0
	Unipolar	11.8	Not assessed	11.6
Lead impedance (Ω)	Bipolar	>3000	510	520
	Unipolar	384	Not assessed	390

## Data Availability

The original contributions presented in this study are included in the article. Further inquiries can be directed to the corresponding author.
